# Bending Angle Sensor Based on Double-Layer Capacitance Suitable for Human Joint

**DOI:** 10.1109/OJEMB.2023.3289318

**Published:** 2023-06-29

**Authors:** Daisuke Goto, Yusuke Sakaue, Tatsuya Kobayashi, Kohei Kawamura, Shima Okada, Naruhiro Shiozawa

**Affiliations:** Graduate School of Sports and Health ScienceRitsumeikan University 12696 Kyoto 603-8577 Japan; Ritsumeikan Global Innovation Research OrganizationRitsumeikan University 12696 Kyoto 603-8577 Japan; Department of RoboticsCollege of Science and EngineeringRitsumeikan University 12696 Kyoto 603-8577 Japan; College of Sports and Health ScienceRitsumeikan University 12696 Kyoto 603-8577 Japan

**Keywords:** Cycling, hysteresis, knee joint, rehabilitation, wearable sensor

## Abstract

*Goal*: To develop bending angle sensors based on double-layer capacitance for monitoring joint angles during cycling exercises. *Methods*: We develop a bending angle sensor based on double-layer capacitive and conducted three stretching, bending, and cycling tests to evaluate its validity. *Results*: We demonstrate that the bending angle sensor based on double-layer capacitance minimizes the change in the capacitance difference in the stretching test. The hysteresis and root mean square error (RMSE) compared with the optical motion capture show hysteresis: 8.0% RMSE and 3.1° in the bending test. Moreover, a cycling experiment for human joint angle measurements confirm the changes in accuracy. The RMSEs ranged from 4.7° to 7.0°, even when a human wears leggings fixed with the developed bending-angle sensor in the cycling test. *Conclusion*: The developed bending angle sensor provides a practical application of the quantitative and observational evaluation tool for knee joint angles.

## I. Introduction

Joint angle monitoring provide doctors and therapists with the benefit of quantitatively and objectively evaluating joint functionality in rehabilitation. Following total knee arthroplasty (TKA) and anterior cruciate ligament (ACL) reconstruction, range of motion (ROM) is essential, particularly in knee flexion recovery [1], [2]. Thus, in the postoperative rehabilitation period, joint angle monitoring is performed to determine the prognosis and efficacy of an intervention [3]. Moreover, the stroke impairment assessment set (SIAS) [4] and Fugl-Meyer motor assessment for lower extremities (FMA-LE) [5] are commonly adopted to evaluate joint function in stroke patients. However, depending on the experience and subjectivity of the therapist, SIAS and FMA-LE may not be used to quantitatively and objectively evaluate the joint functions of a stroke patient [6]. Joint angle monitoring instruments enable us to evaluate joint function quantitatively and objectively in cycling exercises commonly used in the rehabilitation of stroke patients [7], [8]. Moreover, joint angle monitoring instruments may be useful for applications in e-healthcare, athlete training, and biomechanics.

Cameras, including marker-based optical motion capture systems, have been widely used as general evaluation techniques to measure human joint angles, particularly in the field of biomechanics [9]. However, general evaluation techniques require a certain distance from the videos to compute the joint angles [10], [11]. Hence, conventional evaluation techniques face difficulties due to space restrictions on the distance and the need to set the devices in various locations. Stretchable and electrically conductive sensors embedded in wearable garments can provide a solution to space restrictions. Stretchable and electrically conductive sensors enable humans to perform movements to evaluate joint function without space restrictions.

Resistance and capacitive sensors have been developed as electrically conductive stretchable sensors. However, such electrically conductive stretchable sensors have a common problem in that the stretching and bending deformations are simultaneously detected. In previous studies, Lorussi et al. and Tognetti et al. developed bending angle sensors based on double-layer resistance [12], [13]. The output from the bending-angle sensor based on the double-layer resistance remains constant by stretching; however, the bending changes the electrical resistance difference between the two layers. However, Tognetti et al. pointed out that a bending angle sensor based on double-layer resistance still has an angular error due to hysteresis [13]. Hysteresis is a considerable drawback of resistance sensors [14] and makes joint angle estimation difficult because of the non-linear relationship between stretching deformation and resistance [15], [16]. The drawback is that the conductive materials used in resistance sensors undergo irreversible changes during stretching. Consequently, the irreversible change in the conductive material affects the hysteresis behavior [14]. Mengüç et al. [17] reported an error of less than 5° during walking and less than 15° during running using their developed resistance sensor.

The working of capacitive sensors depends on the overlapped area between the electrodes. As a result, the hysteresis on the capacitive sensor is small [18], [19] because the capacitance of a capacitive sensor has a linear relationship with stretching deformation [20]. Furthermore, the electrical stability of the capacitive sensor is superior for repeated stretching and release [21]. However, when the skin touches the capacitive sensors, the measurement accuracy of the joint angles worsens owing to mutual capacitive couplings [22], [23]. Totaro et al. [24] developed a capacitive sensor that shields a two-sided surface; however, the accuracy was not confirmed during walking and running.

Although the aforementioned studies have used bending-angle sensors based on double-layer resistance, there are no reports on bending-angle sensors based on double-layer capacitance. In the present study, the bending angle sensor changes the double-layer resistance into double-layer capacitance to solve hysteresis and cross-sensitivity to stretching. Moreover, for mutual capacitive couplings, shielding a two-sided surface on the bending-angle sensor effectively prevents electromagnetic interference, which is the primary drawback of the capacitive sensor. Using the developed bending-angle sensor based on double-layer capacitance, we demonstrate angle measurement in a broader bending angle range compared to existing studies. Therefore, the novelty of this work is that it aims to develop a bending-angle sensor based on double-layer capacitance for measuring the joint angle during cycling exercise, even when the skin touches the sensors.

## II. Materials and Methods

### A. Principle of System of Bending Angle Sensor Based on Double-Layer Capacitance

**Fig. 1.**
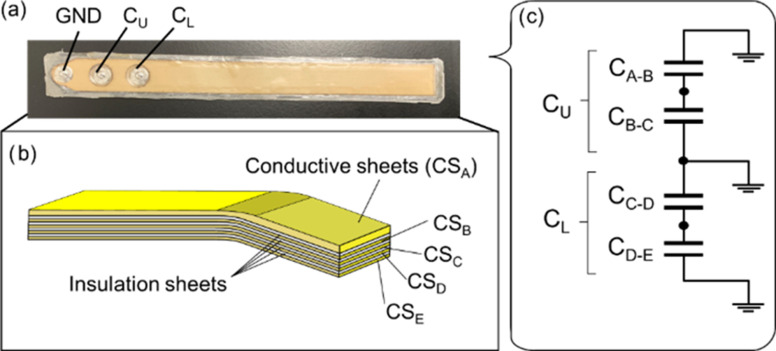
(a) Bending angle sensor based on double-layer capacitance. The ground (GND) was established on the left side of the connections. C_U_ was placed at the middle of the connections, and C_L_ was placed at the right of the connections. (b) Diagram of the bending angle sensor based on double-layer capacitance. Each CSs was overlapped via CS_A_, CS_B_, CS_C_, CS_D_, and CS_E_. The insulation sheets were sandwiched between each CSs. (c) Electrical schematic of the bending angle sensor based on double-layer capacitance. C_U_ and C_L_ comprise parallel connections of CS_A_ to CS_B_ and CS_C_ to CS_D_.

We propose a bending angle sensor (Fig. 1(a)) using five conductive sheets (CSs). As shown in Fig. 1(b), the insulation sheets were sandwiched between the conductive sheets, and then C_A-B_ between CS_A_ and CS_B_, C_B-C_ between CS_B_ and CS_C_, C_C-D_ between CS_C_ and CS_D_, and C_D-E_ between CS_D_ and CS_E_ form the four capacitances. From Fig. 1(c), the combination of C_A-B_ and C_B-A_ is defined as the upper capacitance (C_U_), because C_A-B_ and C_B-A_ are connected in parallel. In addition, the combination of C_C-D_ and C_D-E_ is defined as a lower capacitance (C_L_) because C_C-D_ and C_D-E_ are connected in parallel. Moreover, CS_A_ and CS_B_ cover the bending angle sensor based on double-layer capacitance as electromagnetic interference shielding. Electromagnetic interference shielding facilitated the measurement of the human joint angle using a bending angle sensor based on double-layer capacitance to prevent mutual capacitive coupling with the skin.

**Fig. 2.**
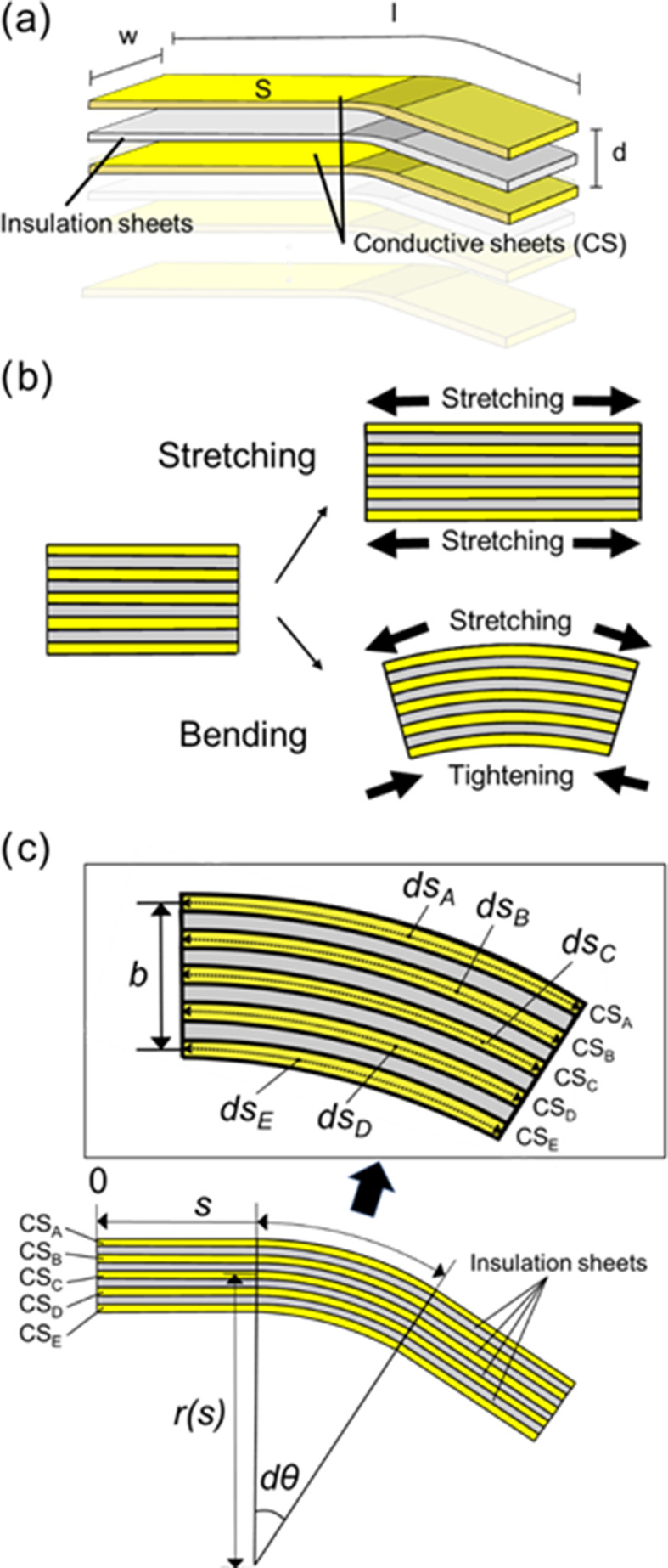
(a) The parameters and capacitances of CSs combined with insulation sheets. (b) Stretch of the bending angle sensor based on double-layer capacitance increases the area of both C_U_ and C_L_. Bending of the bending angle sensor based on double-layer capacitance increases the area of C_U_ and decreases the area of the C_L_. (c) The parameter of each four capacitances in the double-layers capacitance under bending.

As depicted in Fig. 2(b), the electrode area increases when stretching the bending angle sensor based on the double-layer capacitance. This bending angle sensor based on the double-layer capacitance outputs the capacitance difference between C_U_ and C_L_. The capacitance values of the two conductive sheets (Fig. 2(a)) as a single-layer capacitive sensor is calculated using the following equation [18]:

\begin{align*}
\begin{array}{c} {C = {\varepsilon }_0{\varepsilon }_r\frac{S}{d},} \end{array} \tag{1}
\end{align*}

where *C* denotes the capacitance value, ε_0_ indicates the permittivity in a vacuum, ε_r_ refers to the relative permittivity of the insulation sheet, *l* denotes the length of the bending angle sensor based on double-layer capacitance, and *w* denotes the width of the bending angle sensor based on double-layer capacitance. Moreover, *S = lw* yields the area of the overlapped area of the CSs, and d indicates the distance between the CSs. Since (1) is dependent on the overlap area, the overlapped area increases the capacitance when stretched.

Both C_U_ and C_L_ increase, and the capacitance difference between C_U_ and C_L_ remains constant when stretching the bending angle sensor based on the double-layer capacitance. Bending the bending angle sensor based on double-layer capacitance increases the area of C_U_ by stretching the area of C_U_ and decreases the area of C_L_ by tightening the area of C_L_ (Fig. 2(b)). Thus, the bending angle sensor based on double-layer capacitance measures bending but ignores stretching. Moreover, ds_A_, ds_B_, ds_C_, ds_D_, and ds_E_ represent local curves illustrated in Fig. 2(c). When the bending angle sensor based on double-layer capacitance bends, ds_A_, ds_B_, ds_D_, and ds_E_ can be obtained as shown in the following equation:

\begin{align*}
 \left\{ \begin{array}{l} {d{s}_A = \left({r\left(s \right) + \frac{b}{2}} \right) \cdot d\theta }\\ {d{s}_B = \left({r\left(s \right) + \frac{b}{4}} \right) \cdot d\theta }\\ {d{s}_C = r\left(s \right) \cdot d\theta }\\ {d{s}_D = \left({r\left(s \right) - \frac{b}{4}} \right) \cdot d\theta }\\ {d{s}_E = \left({r\left(s \right) - \frac{b}{2}} \right) \cdot d\theta } \end{array}, \right. \tag{2}
\end{align*}

where r(s) represents the curvature and the local central angle is dθ. Equation (2) indicates that ds_A_ and ds_B_ stretches are based on ds_C_, whereas ds_D_ and ds_E_ are tightened based on ds_C_. The length change rate based on ds_C_ is defined as ɛ. Each ɛ represents (3), substituting (2) for ds_A_, ds_B_, ds_C_, ds_D_, and ds_E_ yields:

\begin{align*}
 \left\{ {\begin{array}{l} {{\varepsilon }_A = \frac{{d{s}_A - d{s}_C}}{{d{s}_c}} = \frac{b}{{2r\left(s \right)}}}\\ {{\varepsilon }_B = \frac{{d{s}_B - d{s}_C}}{{d{s}_c}} = \frac{b}{{4r\left(s \right)}}}\\ {{\varepsilon }_D = \frac{{d{s}_D - d{s}_C}}{{d{s}_c}} = - \frac{b}{{4r\left(s \right)}}}\\ {{\varepsilon }_E = \frac{{d{s}_E - d{s}_C}}{{d{s}_c}} = - \frac{b}{{2r\left(s \right)}}} \end{array}} \right., \tag{3}
\end{align*}

where each length change rate ɛ_A_, ɛ_B_, ɛ_D_, and ɛ_E_ is based on ds_C_. Furthermore, each length change Δl_A_, Δl_B_, Δl_D_, and Δl_E_ were calculated by integrating ɛ_A_, ɛ_B_, ɛ_D_, and ɛ_E_. Moreover, Δl_A_, Δl_B_, Δl_D_, and Δl_E_ are expressed as follows:

\begin{align*}
 \left\{ \begin{array}{l} \Delta {l}_A \!=\! \mathop \int \nolimits_0^l {\varepsilon }_Ads \!=\! \mathop \int \nolimits_0^\theta \frac{b}{{2r\left(s \right)}}r\left(s \right)d\theta \!=\! \left[ {\frac{b}{2}\theta } \right]\begin{array}{c} \theta \\ 0 \end{array} \!=\! \frac{b}{2}\theta - 0 \!=\! \frac{b}{2}\theta \\
 \Delta {l}_B \!=\! \mathop \int \nolimits_0^l {\varepsilon }_Bds \!=\! \mathop \int \nolimits_0^\theta \frac{b}{{4r\left(s \right)}}r\left(s \right)d\theta \!=\! \left[ {\frac{b}{4}\theta } \right]\begin{array}{c} \theta \\ 0 \end{array} \!=\! \frac{b}{4}\theta + 0 \!=\! \frac{b}{4}\theta \\
 \Delta {l}_D \!\!=\!\! \mathop \int \nolimits_0^l\! {\varepsilon }_Dds \!=\! \mathop \int \nolimits_0^\theta \!- \frac{b}{{4r(s)}}r(s)d\theta \!\!=\!\! \left[ { - \frac{b}{4}\theta } \right]\!\begin{array}{c} \theta \\ 0 \end{array} \!\!=\!\! - \frac{b}{4}\theta \!+\! 0 \!\!=\!\! - \frac{b}{4}\theta \\ 
\Delta {l}_E \!=\! \mathop \int \nolimits_0^l \!{\varepsilon }_Eds \!=\!\! \mathop \int \nolimits_0^\theta \!- \frac{b}{{2r(s)}}r(s)d\theta \!\!=\!\! \left[ { - \frac{b}{2}\theta } \right]\begin{array}{c} \theta \\ 0 \end{array} \!=\!\! - \frac{b}{2}\theta \!+\! 0\! \!=\!\! - \frac{b}{2}\theta \end{array} \right. \tag{4}
\end{align*}

As mentioned above, the capacitance difference between C_U_ and C_L_ can be obtained from (1) and (4) because Δl_A_, Δl_B_, Δl_D_, and Δl_E_ are the length changes from *l*, which is the length of the bending angle sensor based on double-layer capacitance. Consequently, the capacitance difference between C_U_ and C_L_ is given by the following equation:

\begin{align*}
 \Delta C =& \left({{C}_{A - B} + {C}_{B - C}} \right)- \left({{C}_{C - D} + {C}_{D - E}} \right)\\
 =& \left\{ {{\varepsilon }_0{\varepsilon }_r\frac{{w\left({l + \Delta {l}_A} \right)}}{{\frac{b}{4}}} + \ {\varepsilon }_0{\varepsilon }_r\frac{{w\left({l + \Delta {l}_B} \right)}}{{\frac{b}{4}}}} \right\}\\
& - \left\{ {{\varepsilon }_0{\varepsilon }_r\frac{{w\left({l + \Delta {l}_D} \right)}}{{\frac{b}{4}}} + \ {\varepsilon }_0{\varepsilon }_r\frac{{w\left({l + \Delta {l}_E} \right)}}{{\frac{b}{4}}}} \right\}\\
 \Delta C =& 2{\varepsilon }_0{\varepsilon }_rw\theta \tag{5}
\end{align*}

Equation (5) indicates the capacitance difference between C_U_ and C_L_, which the bending angle sensor based on double-layer capacitance outputs, including the bending angle. From these equations, we theoretically prove that the capacitance difference between C_U_ and C_L_ is linearly related to the bending angle θ, while the bending angle is independent of the length of the bending angle sensor based on the double-layer capacitance.

### B. Fabrication Procedure of the Double-Layer Capacitive Bending Sensor

Regarding the fabrication procedure of the bending angle sensor based on double-layer capacitance, we utilized conductive elastomers (K3B80S, TOYOBO) as the CSs and polyurethane (PU) sheets (MF10F3 3MTS, TOYOBO) as the insulation sheets. CSs were fabricated by mechanically cutting conductive elastomers using a laser engraving machine (Spirit, GCC). The PU sheets with hot-melt material (which melted over 130 °C) on one side were structured to bond the CSs with the insulation sheets. The length of the bending angle sensor based on the double-layer capacitance was 20 cm, and the width of the bending angle sensor based on the double-layer capacitance was 1.8 cm. A fully automatic heat transfer press (HP-84A, HASHIMA) heated 130 °C to melt the hot-melt material and compress the CS and an insulation sheet for 5 s. Once the CS and insulation sheet were compressed, the hot-melted materials were cured after cooling. Finally, five insulation sheets attached to the CSs were compressed for 5 s using a fully automatic heat-transfer press. Three stainless-steel snappers (11.5 mm × 4.5 mm, SEIWA) were contacted and fixed on CS_B_, CS_D_, and ground (GND) (CS_A_, CS_C_, CS_E_) to connect a capacitance detection system via lead wires.

### C. Capacitive Detection System

**Fig. 3.**
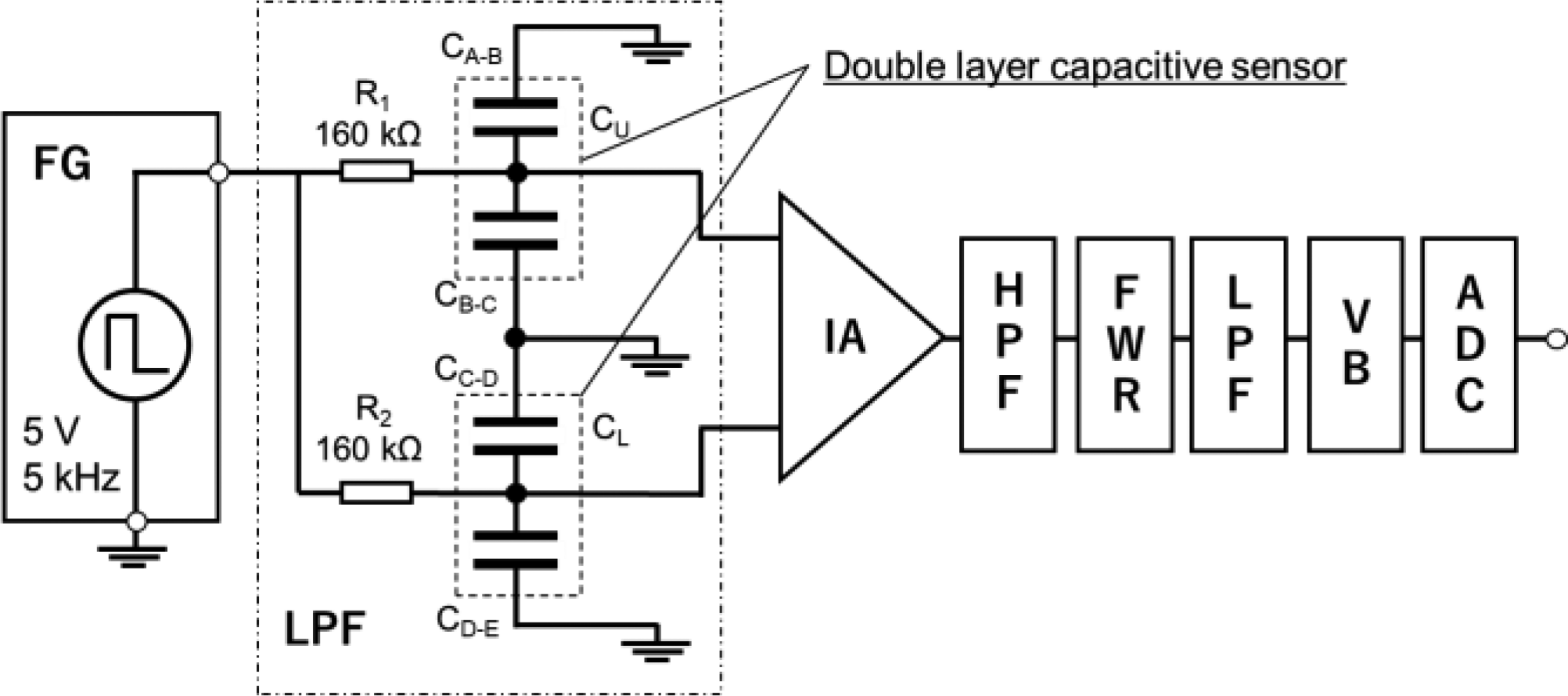
Electrical equivalent of the bending angle sensor based on double-layer capacitance and a block diagram of the capacitance detection system. Function generator (FG), low-pass filter circuit (LPF), instrumentation amplifier (IA), high-pass filter circuit (HPF), full wave rectifier circuit (FWR), voltage buffer circuit (VB), and analog to digital converter.

This section describes the custom-designed capacitance detection system. Fig. 3. illustrates the capacitance detection system for the bending angle sensor based on double-layer capacitance. The capacitance detection system comprises a function generator (AFG-21025, RS PRO), a low-pass filter circuit including a bending angle sensor based on double-layer capacitance, an instrumentation amplifier (AD620ANZ, ANALOG DEVICES), a high-pass filter circuit with a cut-off frequency of 100 Hz, a full-wave rectifier circuit using an operational amplifier (TL071ACP, ANALOG DEVICES), a low-pass filter circuit with a cut-off frequency of 5 Hz, a voltage buffer circuit using an operational amplifier, and an analog-to-digital converter (ADC) (AI-1608AY-USB, CONTEC) with 16-bit resolution. The function generator (AFG-21025, RS PRO) contains a low-pass filter circuit that includes a bending-angle sensor based on double-layer capacitance with a rectangular wave voltage (amplitude = 5 V, frequency = 5 kHz). The low-pass filter circuit comprises a known resistance (R_1_ and R_2_) and capacitance (C_U_ and C_L_). The output voltages of the low-pass filter circuit change with the bending angle because the cut-off frequency of the low-pass filter depends on C_U_ and C_L_. The output voltages of the low-pass filter circuit were amplified, and the output difference was measured using an instrumentation amplifier. In this study, R_1_ and R_2_ were selected as 160 kΩ and employed at the acquired maximum output relative to a specific bending angle. The gain of the instrumentation amplifier was set to 40 dB. The output voltage of the capacitance-detection system was acquired using a PC. The sampling frequency of the capacitance detection system was 100 Hz.

### D. Evaluation Test of C_U_ and C_L_ Value With the Robotic Arm

**Fig. 4.**
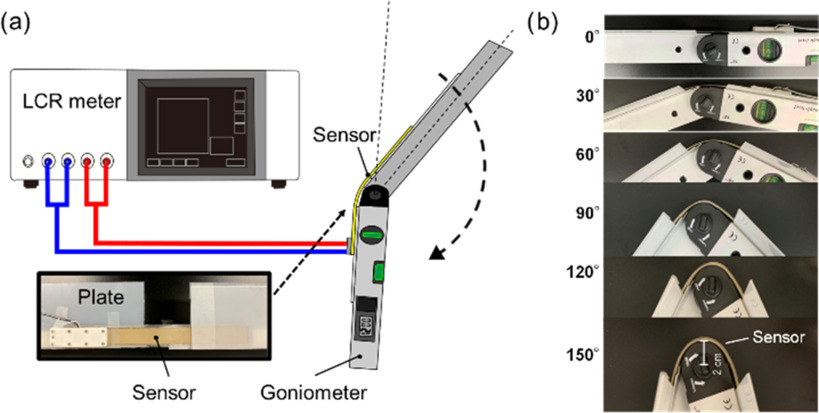
(a) Test system to ensure C_U_ and C_L_ change with the increase of the bending angle. The bending angle sensor based on double-layer capacitance was bent, referring to the goniometer. C_U_ and C_L_ were measured by the chemical impedance analyzer. (b) Bending angle sensor based on double-layer capacitance with the different bending angles (0°–160°) are shown.

We evaluated the bending angle sensor based on the double-layer capacitance without the capacitive detection system to ensure (5) of the measurement principle. C_U_ and C_L_ were measured with an increase in the bending angle of the sensor using a chemical impedance analyzer (IM3590, Hioki) connected to C_U_ or C_L_ via a four-terminal probe. The chemical impedance analyzer was in the range of 100 mΩ ∼ 100 MΩ, and the impedance and phase angle of the accuracy on the chemical impedance analyzer were within the range ±0.05%, ±0.03°, respectively. In the evaluation test, the amplitude and frequency were set to ±2.5 V and 5 kHz, respectively, in the chemical impedance analyzer. We used a goniometer (with an accuracy of ±0.5°, resolution of 0.1°, and circle radius of 20 mm) when a bending angle sensor based on double-layer capacitance was bent. The bending angle sensor based on double-layer capacitance was arranged on the upper part of the goniometer. Moreover, plastic plates on the goniometer fixed both ends of the bending angle sensor based on the double-layer capacitance to bend for the specified accurate angles (Fig. 4(a)). The goniometer bent in the range of 0°–160° in 5° increments (Fig. 4(b)) corresponding to the knee range of motion (typically in the range of 0°–130°). The 20 samples of C_U_ and C_L_ were measured by the chemical impedance analyzer every 5°. The capacitance difference, represented as C_U_–C_L_ was calculated by subtracting C_U_ from C_L_ in (5). The correlation coefficients (R^2^) were calculated by linear regression in least squares on the measured capacitances and bending angles.

### E. Bending and Stretching Test

We conducted a bending test for the bending angle sensor based on double-layer capacitance with an optical motion capture system (V120TRIO, OptiTrack) to evaluate the hysteresis and accuracy of the angle measurement. Both ends of the bending angle sensor based on double-layer capacitance were fixed with tape on two plastic plates to prevent the fixed parts of the bending angle sensor based on the double-layer capacitance from bending. In the bending test, the only part that could bend was the angle sensor based on the double-layer capacitance part without the plastic plate. The part without the plastic plate in the bending angle sensor based on the double-layer capacitance was 3 cm. Furthermore, one end of the bending angle sensor based on the double-layer capacitance in connection with the capacitance detection system was fixed to a desk, and the other remained free to pivot around. The optical motion capture acquired the bending angle of the bending angle sensor based on double-layer capacitance as the reference. The optical motion captured the measured markers accurately within 1.00 mm. As illustrated in Fig. 6(a), the three markers were arranged on both ends and the middle of the bending-angle sensor based on double-layer capacitances. Optical motion capture measured the movement of the three markers on the bending angle sensor based on double-layer capacitance. The vectorial angle θ was calculated as the reference angle using the optical motion capture data that acquired the spatial coordinates of the respective marker positions' x, y, and z. The output voltage of the capacitance detection system was acquired using a PC.

The bending angle sensor based on double-layer capacitance was repeatedly bent by hand in the range of 0–130° for 1 min, referencing the knee range of motion. The repetition speed was adjusted to match the rhythm of an electronic metronome beat of 50 rotations per minute (rpm). Moreover, we conducted robotic arm bending tests to confirm the response at different speeds and the reproducibility of the accuracy Fig. 6(b)). We used Arduino Uno (Arduino, Torino) to rotate the robotic arm automatically at three different speeds (16, 32, and 48 rpm), to emulate for the cycling exercise. The robotic arm could rotate in the range of 20°–120°. The robotic arm bending test was repeated 10 times at different speeds.

**Fig. 5.**
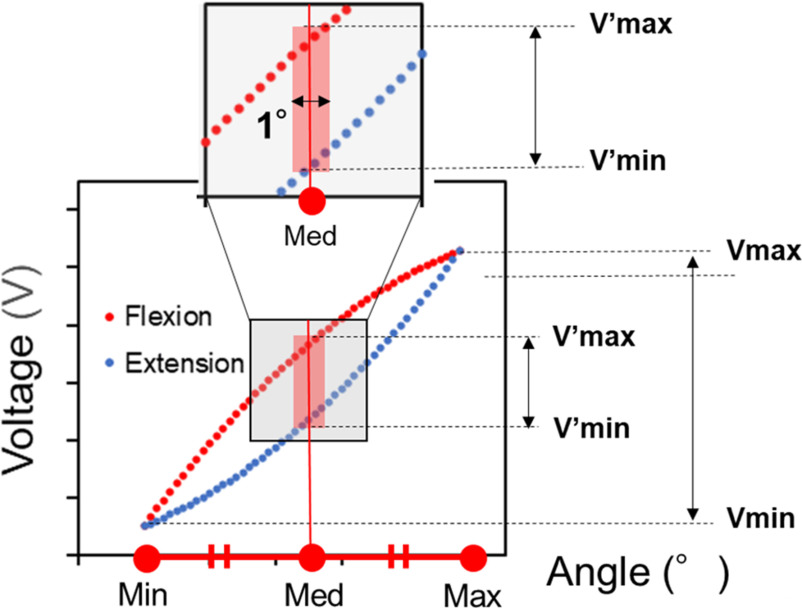
Hysteresis evaluation method. The scatter plot resulted from the measured output voltage with respect to the angle during flexion-extension. The gray region shows the middle part of the scatter plot, including the middle angle. The middle angle is located at the center of the red region. The horizontal distance of the red region is 1°. The vertical distance of the red region is the differential output voltage of ${{\mathrm{V}^{\prime}}}_{\text{max}}$ and ${{\mathrm{V}^{\prime}}}_{\text{max}}$ at the middle angle±0.5°.

Linear regression in least squares was performed on the output voltage of the bending-angle sensor based on the double-layer capacitance and the reference angle. The estimated angle and R^2^ were calculated using the linear regression equation of the reference angle to the output voltage of the bending angle sensor based on the double-layer capacitance. The root mean square error (RMSE) was calculated from the error between the estimated and reference angles. Furthermore, Fig. 5 shows the hysteresis evaluation method. The middle angle is the midpoint between the maximum and minimum angles. The maximum and minimum output voltages at the middle angle ±0.5° are represented as ${{\mathrm{V}^{\prime}}}_{\text{max}}$ and ${{\mathrm{V}^{\prime}}}_{\text{max}}$, respectively [25]. In Fig. 5, the gray region represents as the middle part, and the vertical distance of the red region indicates the differential output voltages of ${{\mathrm{V}^{\prime}}}_{\text{max}}$ and ${{\mathrm{V}^{\prime}}}_{\text{max}}$. The maximum and minimum output voltages in the bending angle range in the test are donated as V_max_ and V_min_, respectively. Hysteresis is defined as the ratio of the differential output voltage of ${{\mathrm{V}^{\prime}}}_{\text{max}}$ and ${{\mathrm{V}^{\prime}}}_{\text{max}}$ to the difference between V_max_ and V_min_ in the bending test. Thus, the hysteresis was evaluated using the following formula:

\begin{align*}
 Hysteresis = \ \frac{{{{V^{\prime}}}_{max} - {{V^{\prime}}}_{min}}}{{{V}_{max} - {V}_{min}}} \times 100 \tag{6}
\end{align*}

**Fig. 6.**
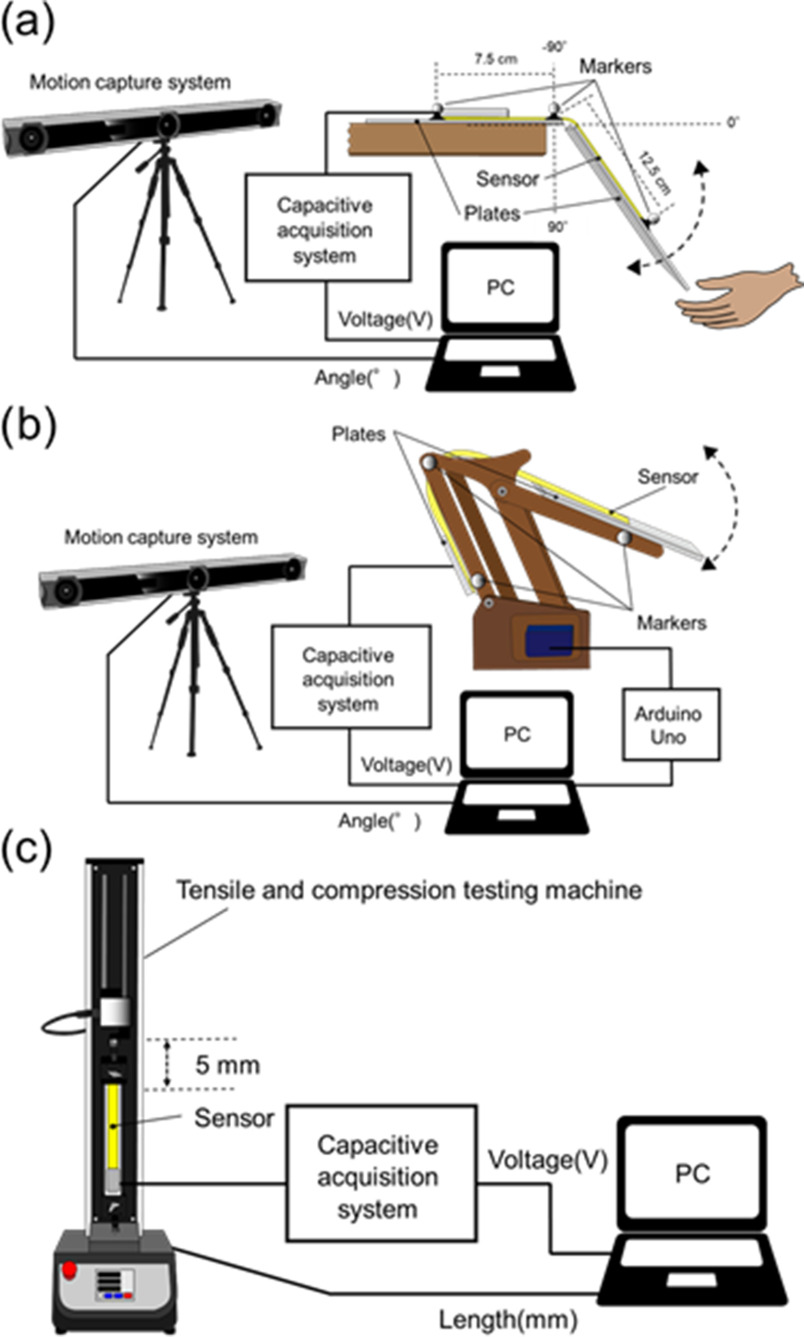
(a) Test system used for the bending test. (b) Test system used for the robotic arm bending test. The optical motion capture acquired the three markers arranged on the bending angle sensor based on double-layer capacitance, and the capacitance detection system simultaneously measured the output voltage of the bending angle sensor based on double-layer capacitance. (c) Test system used for the stretching test. When the desktop tensile compression tester stretched the bending angle sensor based on double-layer capacitance and the bending angle sensor based on single-layer capacitance, the output voltages of both the bending angle sensor based on double-layer capacitance and the bending angle sensor based on single-layer capacitance were acquired.

Subsequently, we conducted a stretching test for the bending angle sensor based on double-layer capacitance in addition to a bending angle sensor based on single-layer capacitance to ensure that the difference between C_U_ and C_L_ is maintained. The output voltages of stretching were investigated for the bending angle sensor based on the double-layer capacitance and the bending angle sensor based on the single-layer capacitance (Fig. 6(c)). As mentioned in Section II, the bending-angle sensor based on single-layer capacitance responds to both stretching and bending, because the overlap area of the CSs increases. Therefore, a bending angle sensor based on single-layer capacitance was prepared in the stretching test for comparison with the bending angle sensor based on double-layer capacitance. A desktop tensile compression tester (MCT-2150, A&D) stretched the bending angle sensor based on double-layer capacitance and a bending angle sensor based on single-layer capacitance clamped at both ends using metal plates and screws. The desktop tensile compression tester performed five stretching/tightening cycles with a total stretch of 5 mm, at a speed of 10 mm/min. We acquired the output voltages of the bending angle sensor based on the double-layer capacitance and the bending angle sensor based on single-layer capacitance via a capacitance detection system. Moreover, we obtained the length change from a desktop tensile compression tester.

### F. Knee Joint Angle Measurement in the Cycling Experiment

We evaluated the accuracy of the knee bending angle for the bending angle sensor based on double-layer capacitance when a human wearing the wearable garment fixed the bending angle sensor based on double-layer capacitance and performed a high-intensity knee flexion-extension movement, such as cycling exercise, to demonstrate the validity of shielding a two-sided surface on the bending angle sensor based on double-layer capacitance. A healthy subject (age 28 years, weight 56.5 kg, height 162.5 cm) without a history of pain and disability of the hip and knee performed the cycling exercise while wearing the legging with the fixed bending angle sensor based on double-layer capacitance. Prior to the test, informed consent was obtained from all the subjects. The experiment conformed to the standards of the declaration of Helsinki and was approved by the Ethics Committee of Ritsumeikan University (BKC-LSMH-2021-043). In this experiment, the focus was on the cycling exercise, which has a lower risk of falling, smaller knee stresses, and more bending knee joints than gait exercise. The cycling exercise is suitable for the rehabilitation of stroke patients to monitor the knee joint angle and improve locomotor and cardiorespiratory functions [7], [8].

**Fig. 7.**
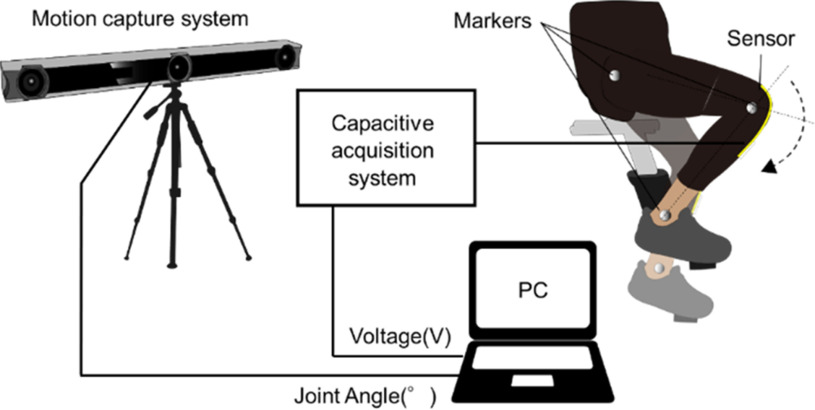
Test system of the knee angle measurement during the cycling exercise.

The subject performed cycling at submaximal intensities for 10 min, adjusting the knee flexion-extension speed at 30 rpm using an electronic metronome. The double-layer capacitive bending was attached to a legging (MCM8858, UNDER ARMOUR) by sewing with a needle and thread. The subject wore the leggings and adjusted their position on the knee joint, as illustrated in Fig. 7. The output voltage of the bending angle sensor based on double-layer capacitance was recorded for 10 min by a PC using a capacitance detection system fixed on a desk. The optical motion capture recorded the knee flexion-extension angle as the reference angle ten times for 30 s every minute from the beginning of the cycling exercise. The three markers were anchored to the greater trochanter, lateral epicondyle of the femur, and lateral malleolus of the ankle on the right side of the subject. We calculated the vector angle θ as the reference angle from the three markers that formed the straight lines. Subsequently, the acquired output voltage of the bending angle sensor based on the double-layer capacitance was separated into ten output voltages. Linear regression in least squares was performed on each separated output voltage of the bending angle sensor based on double-layer capacitance and ten reference angles for 30 s, and each of the estimated angles, R^2^, and RMSE were calculated. Furthermore, the slopes of the baselines of the estimated angles were calculated using linear least-squares regression and the angles for all 10 min datasets and 10 separate 30 s datasets obtained using the bending angle sensor based on double-layer capacitance were estimated. The slopes of the baselines of the reference angles were also calculated using linear least-squares regression, and the results were compared with the calculated slopes of the estimated angles.

## III. Results

### A. Evaluation Test of C_u_ and C_l_ Value With the Robotic Arm

**Fig. 8.**
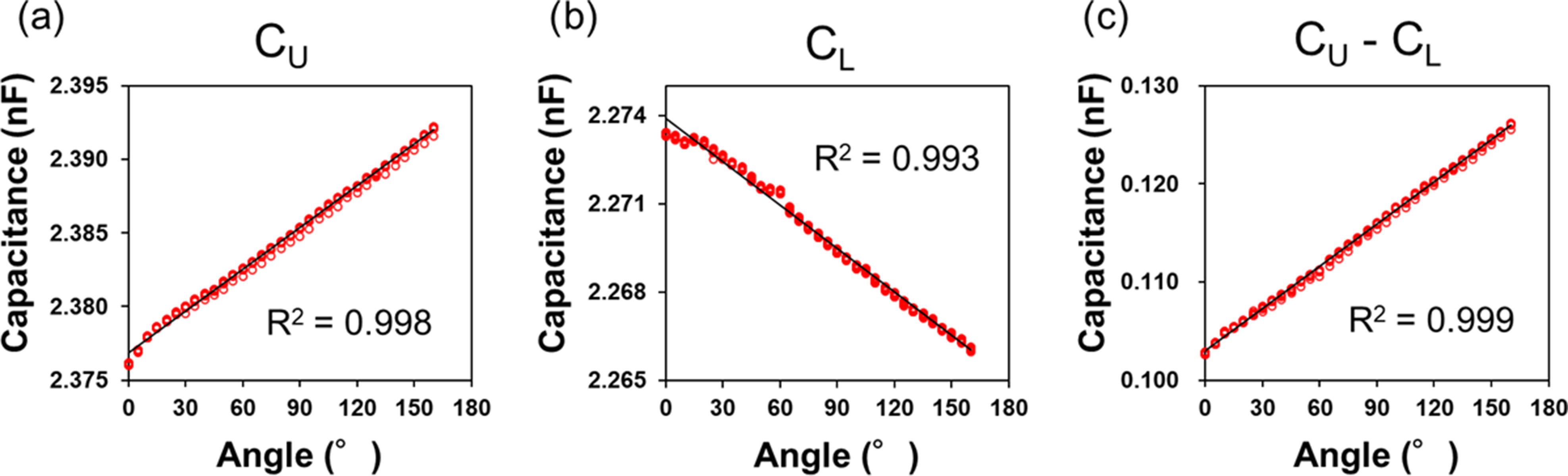
Correlation plots between the capacitances ((a) C_U_, (b) C_L_, and (c) C_U_–C_L_) measured by the chemical impedance analyzer and the bending angle measured by the goniometer. C_U_–C_L_ was calculated by subtracting C_U_ from C_L_.

Fig. 8 illustrates the capacitances (a: C_U_, b: C_L_, and c: C_U_-C_L_) measured by the chemical impedance analyzer to the bending angle referencing the goniometer. The capacitive change of C_U_ increased linearly with the bending angle (R^2^ = 0.998), whereas the capacitive change in C_L_ decreased linearly with the bending angle (R^2^ = 0.993), as described in Section II. Furthermore, the capacitive change in the C_U_-C_L_ was more linearly proportional to the bending angle (R^2^ = 0.999), as depicted in Fig. 8(c).

### B. Bending and Stretching Test

**Fig. 9.**
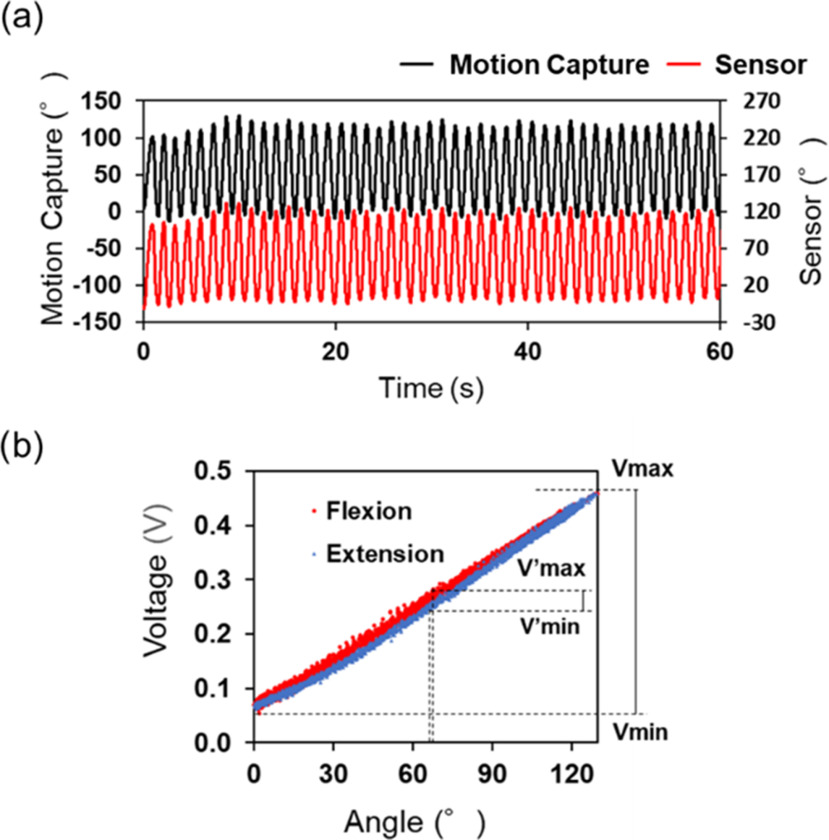
(a) Dynamic response comparison between the bending angle sensor based on double-layer capacitance and optical motion capture for 1 min. The black line represents the knee angle of the optical motion capture, and the red line represents the estimated knee angle of the bending angle sensor based on double-layer capacitance. (b) Hysteresis curve of the bending angle sensor based on double-layer capacitance (hysteresis = 8.0%). The red plots represent flexion, and the blue plot represents extension. The maximum output voltage is within 0°∼130° (V_max_), minimum output voltage is within 0°∼130° (V_min_), maximum output voltage is within ±65° ${\mathrm{(}}{{\mathrm{V}^{\prime}}}_{\text{max}}{\mathrm{)}}$, the minimum output voltage within ±65° $({{{{\mathrm{V}^{\prime}}}}_{\text{min}}})$).

Fig. 9(a) presents the dynamic response comparison between optical motion capture and the bending angle sensor based on double-layer capacitance. We observed that these dynamic responses were similar in the bending test. The hysteresis and RMSE of the bending angle sensor based on double-layer capacitance was 8.0% and 3.1° within 0° ∼ 130°, based on the reference angle. Fig. 9(b) illustrates the hysteresis curve of the bending angle sensor based on double-layer capacitance based on the reference data. There is great agreement between the output behavior of the flexion (red plots) and the extension (blue plots). Moreover, R^2^ of the output voltage and the reference data was 0.996, indicating the high linearity.

**Fig. 10.**
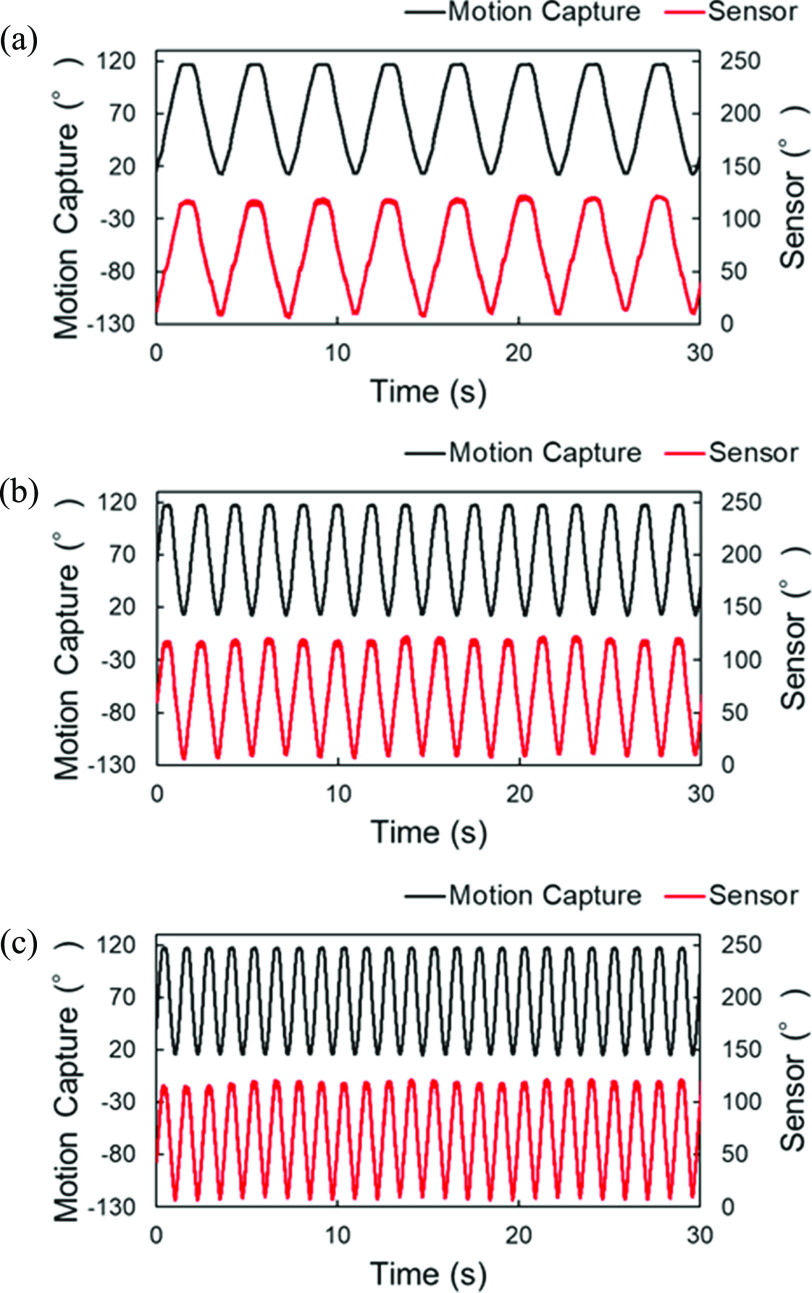
Dynamic response comparisons between the bending angle sensor based on double-layer capacitance and optical motion capture for 30 s at (a) 16 rpm, (b) 32 rpm, and (c) 48 rpm.

Fig. 10 compares the results for each representative dynamic response obtained over 30 s in the robotic arm bending tests. The output voltages from the bending angle sensor based on the double-layer capacitance could respond to the movement of the robotic arm. The results for the hysteresis (based on all the trials), RMSE, and R^2^ at 16 rpm were 7.5 ± 1.0%, 2.9 ± 0.2°, and 0.994 ± 0.001, respectively. Similarly, the hysteresis, RMSE, and R^2^ based on all the trials at 32 rpm were 7.7 ± 0.7 %, 3.0 ± 0.1°, and 0.994 ± 0.001, respectively. The RMSE and R^2^ values based on all trials were 3.5 ± 0.2 and 0.991 ± 0.001 at 48 Hz, respectively. The hysteresis at 48 rpm was 10.0 ± 1.2 %.

**Fig. 11.**
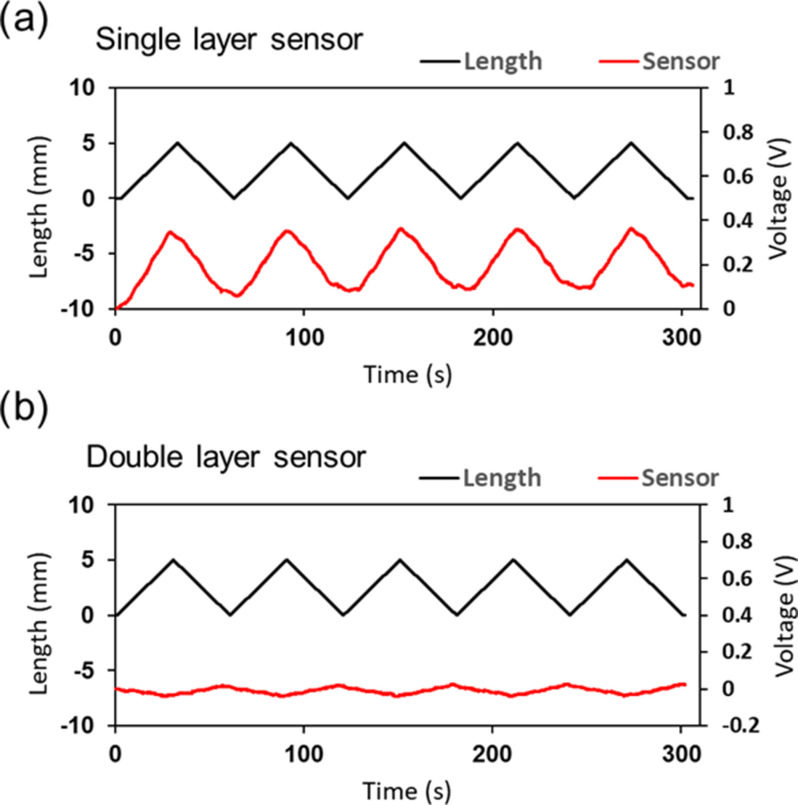
(a) Dynamic response comparison between the output voltage and the length change in the stretching test with a total stretch of 5 mm at a speed of 10 mm/min. Bending angle sensor based on (a) single-layer capacitance, and (b) double-layer capacitance.

Fig. 11 shows the dynamic response of the single-layer capacitive sensor (Fig. 11(a)) and the bending angle sensor based on double-layer capacitance (Fig. 11(b)) obtained using the desktop tensile compression tester. The estimated angle of the bending angle sensor based on single-layer capacitance was affected from −0.4° to 54.4°, whereas the estimated angle of the bending angle sensor based on double-layer capacitance was affected from −9.2° to 9.8°. The bending angle sensor based on double-layer capacitance reduced the estimated angle error under stretching by 65%. The results of the stretching test indicate that the bending-angle sensor based on the double-layer capacitance minimized the output voltage change under stretching.

### C. Knee Joint Angle Measurement in the Cycling Experiment

**Fig. 12.**
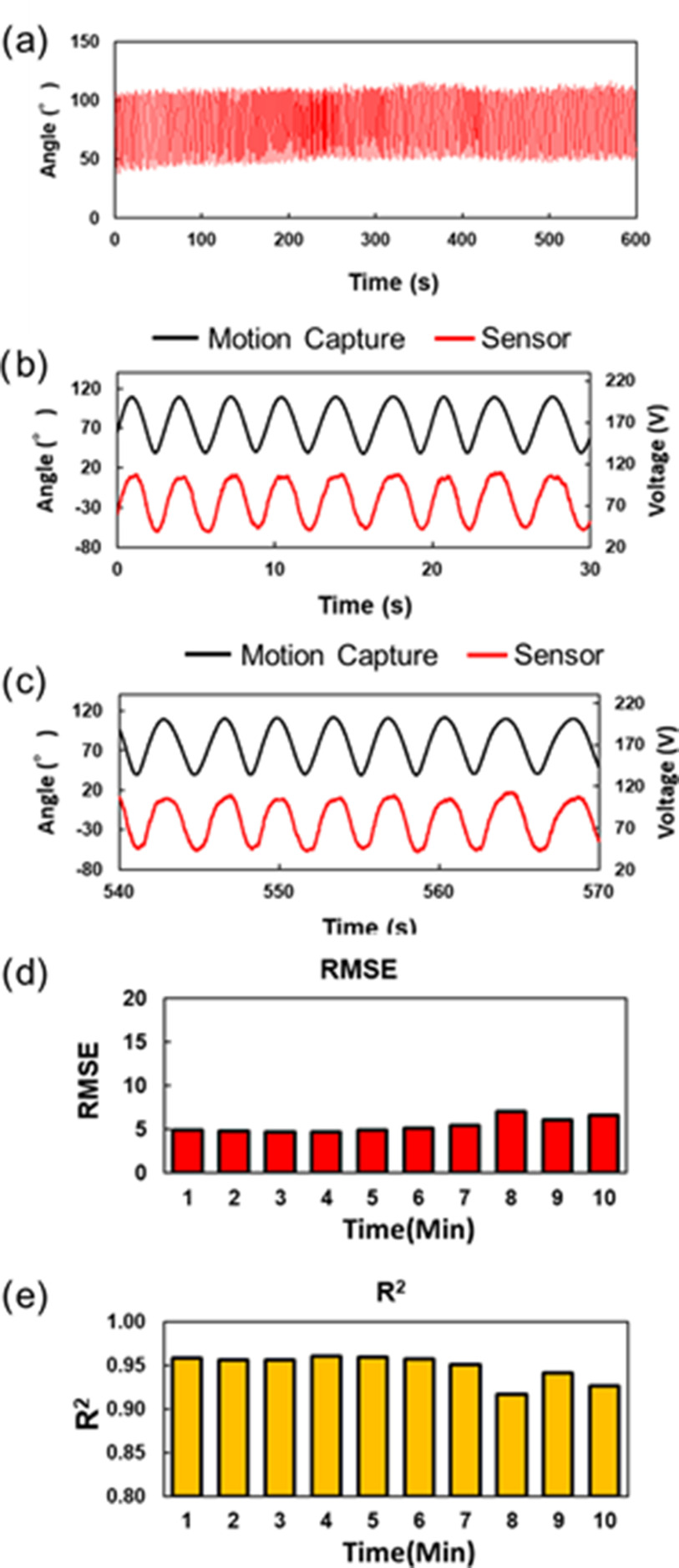
(a) Result of the estimated knee angle measurement during the cycling exercise at 30 rpm for 10 min. The dynamic response comparison between the bending angle sensor based on double-layer capacitance and the optical motion capture for (b) 30 s after the start and (c) 30 s before the finish. The black line represents the knee angle of the optical motion capture, and the red line represents the output voltage of the bending angle sensor based on double-layer capacitance. (d) Trends in time of RMSEs. (e) Trends in time of R^2^.

Fig. 12(a) illustrates the estimated angle of the bending angle sensor fixed on the leggings, which is based on double-layer capacitance. Although the knee flexion-extension motion may cause spike noises owing to excessive tensions around the connectors, noise spikes and large amplitude changes were not observed, even during the cycling exercise. Fig. 12(b) and (c) present the output voltage of the bending angle sensor based on double-layer capacitance and the reference angle for 30 s after the start and before the finish. Great repeatability and similarity were observed for both results.

Figs. 10(e) and 12(d) show fluctuations of RMSE and R^2^ during cycling exercise for 10 min. The RMSE ranged from 4.7° to 7.0°, and R^2^ ranged from 0.917 to 0.960, maintaining high linearity when a human wore the leggings. RMSE and R^2^ highlighted the high accuracy of the bending angle sensor based on double-layer capacitance.

Another concern is the slow drift, which modifies the capacitance difference between C_U_ and C_L_. However, there was negligible drift (slope < 0.01°) for all 10 min data throughout the cycling exercise. Table I shows the calculated slopes of the baselines for the estimated angles and the reference angles for 10 separate 30 s datasets. The maximum slope of the estimated angle was 0.30° at 9 min, and the slope of the reference angle was 0.26° at 9 min. The minimum slopes of the estimated and reference angles were 0.21° and 0.26° at 8 min, respectively.

**TABLE I:** Slopes Calculated for 10 Separate 30 s Datasets During the Cycling Exercise

Time (Min)	Bending Angle Sensor (°)	Motion Capture (°)
1	-0.07	-0.18
2	0.05	-0.04
3	0.09	0.06
4	0.08	0.10
5	-0.14	-0.13
6	-0.02	0.03
7	-0.02	0.01
8	-0.21	-0.20
9	0.30	0.26
10	<0.01	-0.02
Average	<0.01	-0.01
SD	0.13	0.13

## IV. Discussion

A bending angle sensor based on double-layer capacitance was developed to solve the problems of hysteresis and simultaneous detection of stretching and bending. We conducted tests on the bending angle sensor. Subsequently, we demonstrated that knee joint angle monitoring using a bending angle sensor based on double-layer capacitance is useful in cycling exercises.

Fig. 8 shows that the bending angle sensor based on double-layer capacitance increases the area of C_U_ and decreases the area of C_L_ when bending, so that the capacitance difference between C_U_ and C_L_ is significantly related to the bending angle based on (5). This means that the capacitance difference between C_U_ and C_L_ is proportional to the angle. In similar studies, Totaro et al [24] and Nakamoto et al [26] confirmed the linearity of capacitance sensors in tensile tests instead of bending tests. In other words, it is elongation, not bending, that is being measured, and it is unclear whether the relationship with angle is linear. Also, Li et al. reported a quadratic relationship between the capacitance of capacitive bend angle sensors and the bend angle at calibration [27]. The fact that the relationship between angle and sensor output is expressed as quadratic means that it is difficult for this sensor to detect any angle as a uniform sensitivity. For the sensor we developed, the relationship can be expressed as linear (C_U_: R^2^ = 0.998; C_L_: R^2^ = 0.993), because it is capable of detecting with uniform sensitivity for any angle [12]. In conclusion, we confirm that bending angle sensors based on double-layer capacitance can measure bending angles more accurately than other single-layer capacitance sensors by calculating the difference between C_U_ and C_L_.

The hysteresis and RMSE are compared with the optical motion capture in Fig. 9 were 8.0 %, 3.1 in the bending test, respectively. In addition, the robotic arm bending test demonstrated that the bending angle sensor based on double-layer capacitance could respond at different speeds (Fig. 10) and had a reproducible accuracy. Tognetti et al. reported that a bending angle sensor based on double-layer resistance exhibited different behaviors between flexion and extension [13]. Comparison of the scatter diagrams in Fig. 9(b) and [13] clearly showed that the hysteresis of the bending angle sensor based on double-layer capacitance was reduced compared to that of the bending angle sensor based on double-layer resistance. The capacitance of capacitive sensors depends on the overlap area. Accordingly, capacitive sensors can reduce hysteresis, regarded as a problem with resistive sensors [18], [19]. The bending angle sensor based on double-layer capacitance overcame the mutual capacitive couplings, considered an important obstacle in capacitive sensors [22], [23], by shielding using a two-sided surface [24]. These improvements resulted in the high accuracy and small hysteresis of the bending angle sensor based on the double-layer capacitance in the bending test.

Fig. 11 illustrated that the bending angle sensor could minimize the change in the capacitive difference in the stretching test. According to (5), the capacitance difference between C_U_ and C_L_ remains constant when the bending angle sensor is based on double-layer capacitance stretching. Thus, theoretically, the output voltage of the bending angle sensor based on the double-layer capacitance does not change [13]. However, the output voltage of the bending angle sensor based on the double-layer capacitance changed from −9.2° to 9.8° and exhibited an anti-phase relationship with the length change in the stretching test. We assume that this factor of the output voltage change is a tiny area difference between the C_U_ and C_L_ in the fabrication of the bending angle sensor based on double-layer capacitance [18]. It is possible that the change in output voltage occurred as the overlapped area of the C_L_ became larger than that of the C_U_ under slight stretching.

As illustrated in Fig. 12, the RMSEs ranged from 4.7° to 7.0° in the cycling experiments for the human joint. The RMSE and R^2^ in the cycling experiment were slightly lower in accuracy than those in the bending test because the attachment positions of the bending angle sensor based on double-layer capacitance on the patella differed from the anchored marker positions [17], [26], [28]. The output voltages of the bending angle sensor based on double-layer capacitance delayed the cycles. The RMSE and R^2^ slightly decreased at 8 min, indicating an improvement for a suitable attachment to the wearable garments. Future work will include seeking suitable attachments for complex motion measurements. For example, thin non-slippery pads can tighten the sensor attachments to prevent the slippage of sensors attached on the skin [17]. Alternatively, compensation algorithms including detailed models of bone and muscle shapes could be used to overcome this issue [26].

The slope of all 10-min data proves that the slow drift of the capacitance difference between C_U_ and C_L_ could be ignored, but some slopes of the separate 30 s datasets were large (Table I). However, some large slopes of the reference angle were observed, simultaneously with large slopes on the estimated angle. When the slope of the estimated angle was small (within ±0.05), the slopes of the reference angle fit within ±0.05. The results indicated that the knee joint angle affected some large slopes on the estimated angle. Therefore, the bending angle sensor based on double-layer capacitance demonstrated high accuracy in measuring the knee joint angle [29].

The results of the cycling exercises are shown in Fig. 12, shielding a two-sided surface on a bending angle sensor based on double-layer capacitance demonstrated the prevention of mutual capacitive couplings with the skin. The addition of CS_A_ and CS_E_ as GND prevents electromagnetic interference [24] and stable capacitance measurement because of the increase in the capacitance of C_U_ and C_L_ by the parallel connection. The cycling experiment confirmed the necessity of shielding when measuring human motion using a double-layout capacitive bending sensor.

Table II compares the type of stretchable and electrically conductive sensors to emphasize the benefits of the bending angle sensor based on double-layer capacitance. Although Mengüç et al. [17] and Tognetti et al. [13] reported that resistance sensors exhibited hysteresis, The bending angle sensor based on double-layer capacitance minimized the hysteresis in the bending test. Furthermore, the capacitance difference of the double-layer capacitive sensor could be maintained during stretching, compared to the operating principle of other sensors [17], [24], [27]. The experiments in this study highlight that we performed a broader bending angle range in the bending test and cycling experiment, comparing other studies in static and dynamic experiments. Evidently, the knee joint angle is more extensive in the cycling leg test than during walking [17], [24], [27], [30] and running [17], [24]. Additionally, our cycling experiment continuously recorded knee joint angles for longer than other studies [17], [24], [27] and evaluated the drifts for separate 30 s datasets and all 10 min of data, and demonstrated that the drift was negligible. It should be emphasized that the bending angle sensor based on double-layer capacitance exhibited high accuracy, despite the broader bending angle range and recording longer time.

**TABLE II:** Comparison Between Different Types of Stretchable and Electrically Conductive Sensors

Reference	Type of sensor	Hysteresis	Detection of only bending	Sensor position	Evaluation metrics(Static test)	Evaluation metrics (Dynamic test)
Mengüç et al. [17]	Resistive			Hip, knee, and ankle	Electrical hysteresis percentages (and linearity ratios) of 7.8% (0.77 linearity), 3.9% (0.91), and 4.3% (0.92) for the hip, knee, and ankle (mechanical loading and unloading)	RMSE < 5° (Walking at 0.89 m/s for 60 s) RMSE < 15° (Running at 2.7 m/s for 60 s)
Oubre et al. [30]	Resistive (potentiometer)			Knee		RMSE = 5.0 ± 1.0° MAE = 3.9 ±0.8° (Walking at three different self-selected speeds)
Wood et al. [28]	Resistive			Knee		The average RMSE = 7.6° (Knee flexion-extension movements and walking)
Tognetti et al. [13]	Resistive (Double-layer)		✓	Knee	Maximum standard deviation = 5100 Ω, corresponding to an angular error of 5.3° (quasi-static flexion test in the 0–90° range)	Maximum error = 5.0° (Knee flexion-extension movements)
Nakamoto et al. [26]	Capacitive	✓		Knee and ankle	R^2^ = 0.9993 (stretching test in the 30–70 mm range)	Mean error = 4.4 ± 3.6° (knee flexion-extension movements)
Li et al. [27]	Capacitive	✓		Ankle	R^2^ = 0.9991 (bending test in the 0–90° range)	RMSE < 3.0° (Horizontal and descending walking for 90 s) RMSE < 5.0° (Ascending walking for 90 s)
Totaro et al. [24]	Capacitive (Shielding)	✓		Knee and ankle	Linear behavior in the entire range (tensile test)	RMSE < 4.0° (Ten knee flexion/extension in the 0–90° range)
This work	Capacitive (Double-layer and shielding)	✓	✓	Knee	RMSE = 3.1° (bending test in the 0–130° range) RMSE = 2.9 ± 0.2° (robotic arm bending test in the 20–120° range at 16 rpm) RMSE = 3.0 ± 0.1° (robotic arm bending test in the 20–120° range at 32 rpm) RMSE = 3.5 ± 0.2° (robotic arm bending test in the 20–120° range at 48 rpm)	RMSE < 7.0° (Cycling experiment for 10 min)

It is well known that temperature and humidity affect the dielectric constant. Our proposed sensor, due to its structure, is expected to be less affected by temperature and humidity. Because, assuming that the influence values due to temperature and humidity superimposed on C_U_ and C_L_ are comparable, the influence values of temperature and humidity are expected to cancel out because the difference between C_U_ and C_L_ is calculated. However, this was not proved in this experiment. This needs to be verified in future studies.

The CSs in the bending angle sensor based on double-layer capacitance do not hinder movement because of the stretchable materials. Furthermore, the feature of the bending angle sensor based on double-layer capacitance can be easily attached to a wearable garment. Therefore, the bending angle sensor based on double-layer capacitance can be used easily in rehabilitation and comfortably monitors the joint angle of stroke patients. Rehabilitation monitoring using a bending angle sensor based on double-layer capacitance is expected to provide quantitative and objective joint function evaluations and contribute to determining the prognosis and efficacy of an intervention in the post-operative rehabilitation period.

## V. Conclusion

Joint angle monitoring is variable, particularly during the postoperative rehabilitation period. Although cameras, including marker-based optical motion capture systems, can measure with high accuracy, some limitations of use include space restrictions for the distance and the requirement to set the devices in various locations. Stretchable and electrically conductive sensors embedded in wearable garments can be used to overcome these limitations. However, conventional stretchable and electrically conductive sensors face some difficulties with hysteresis and the simultaneous detection of stretching and bending. Therefore, we developed a bending angle sensor based on double-layer capacitance that can maintain the output constant by stretching and acquiring the change in the electrical capacitance difference between C_U_ and C_L_ by bending. In this study, a bending angle sensor based on double-layer capacitance was fixed on the leggings, and the knee joint angle was measured during the cycling exercise. In the stretching and bending tests, we ensured a constant difference under stretching and a hysteresis behavior under bending. We demonstrated that the bending-angle sensor based on double-layer capacitance can effectively minimize the change in capacitance difference in the stretching test. Moreover, the hysteresis and RMSE based on optical motion capture were small in the bending test. Furthermore, the result of the cycling experiment exhibited a low RMSE even when the human wears the leggings fixed with the bending-angle sensor based on double-layer capacitance. The results show that shielding a two-sided surface on the bending angle sensor based on double-layer capacitance enabled joint angle monitoring during the cycling exercise. Consequently, the new bending-angle sensor based on double-layer capacitance provides practical applications of quantitative and observational evaluation tools for knee joint functions. In future work, we will seek suitable attachments to the bending-angle sensor to perform complex motion measurements.
